# Clinical impact of pulmonary hypertension on the outcomes of acute myocardial infarction patients with or without chronic obstructive pulmonary disease

**DOI:** 10.1097/MD.0000000000028627

**Published:** 2022-01-21

**Authors:** Seok Oh, Ju Han Kim, Kyung Hoon Cho, Min Chul Kim, Doo Sun Sim, Young Joon Hong, Youngkeun Ahn, Myung Ho Jeong

**Affiliations:** Department of Cardiology, Chonnam National University Hospital, Gwangju, South Korea.

**Keywords:** chronic obstructive, hypertension, mortality, myocardial infarction, pulmonary, pulmonary disease

## Abstract

Acute myocardial infarction (AMI) and chronic obstructive pulmonary disease (COPD) are leading global causes of morbidity and mortality. In patients with both of these conditions, the presence of pulmonary hypertension (PH) can further worsen their prognosis. We examined the outcomes of AMI patients with COPD (AMI+COPD) and without COPD (AMI−COPD), depending on the presence or absence of PH.

A total of 318 AMI patients with COPD (AMI+COPD cohort) (n = 109) or without COPD (AMI-COPD cohort) (n = 209) were included in this study and were subdivided into 2 groups according to right ventricular systolic pressure (RVSP) level (PH group [RVSP ≥35 mm Hg] and no PH group [RVSP <35 mm Hg]).

We investigated characteristics and clinical outcomes in both the AMI-COPD and AMI+COPD cohorts. When investigating in-hospital clinical outcomes, the PH group had a higher proportion of new-onset heart failure (HF) in both cohorts. In the AMI+COPD cohort, however, the PH group had a higher incidence of cardiogenic shock than the no PH group, which was consistent with the result of the post-inverse probability of treatment weighting (IPTW) analysis. When investigating 1-year clinical outcomes, the PH group had higher incidences of a major adverse cardiac event and all-cause mortality in both cohorts. This finding was mainly driven by cardiac death in the AMI-COPD cohort, whereas it was mainly driven by non-cardiac death in the AMI+COPD cohort. After IPTW adjustment, these differences were statistically attenuated such that all variables were similar between both groups.

PH may be associated with the development of new-onset HF (in all patients) and cardiogenic shock (in the AMI+COPD cohort). In addition, PH may be also associated with all-cause mortality, although it was statistically attenuated after IPTW adjustment.

## 1 Introduction

Coronary artery disease continues to be recognized as the leading cause of cardiovascular deaths worldwide.^[1,2]^ In particular, acute myocardial infarction (AMI) is considered a medical emergency requiring a prompt revascularization strategy.^[3]^ Because of the increasing trend in the number of survivors of AMI, this disease has become a leading cause of disability-adjusted life-years.^[4]^ Its prevalence appears to rise with early diagnosis and adequate treatment.^[5]^ Nonetheless, a considerable number of these survivors develop ischemic cardiomyopathy (ICM) with progressive deterioration of left ventricle function, intermittently experiencing the clinical symptoms or signs of heart failure (HF).

Pulmonary hypertension (PH) is a clinical condition characterized by an increase in pulmonary artery pressure (PAP).^[6]^ PAP increases the after load of the right ventricle and causes right ventricular (RV) hypertrophy, leading to pathological processes such as mal adaptation, RV dilation, and progressive RV dysfunction.^[7]^ PH is diagnosed as the presence of a mean PAP ≥25 mm Hg at rest when measured by right heart catheterization (RHC),^[7]^ although patients who suffer from exertional dyspnea, syncope, and/or clinical signs of RV dysfunction can also be assessed for suspected PH using transthoracic echocardiography (TTE).^[7–9]^

PH can be induced by left-sided HF or left heart disease, such as AMI and ICM, because pulmonary artery end-diastolic pressure is reasonably related to left ventricular (LV) end-diastolic pressure and left atrial mean pressure.^[10]^ This kind of PH, also known as group 2 PH, is the most prevalent form.^[11,12]^ Its clinical significance in AMI has been mentioned in several articles,^[13,14]^ implicating that PH, manifested by increased RV systolic pressure (RVSP), is associated with an increased mortality^[13]^ and can be a useful predictor for the development of overt HF in AMI.^[14]^

Chronic obstructive pulmonary disease (COPD), a progressive and incurable disease, is one of the leading global causes of morbidity and mortality.^[15,16]^ It is characterized by persistent respiratory symptoms with limitation of airflow, originating from airway and/or alveolar structural abnormalities. Meanwhile, PH is also considered to be one of the most frequent complications of COPD. However, approximately 1% to 5% of patients with COPD have a mean PAP ≥35–40 mm Hg at resting state.^[17]^

Thus, it is easy to imagine that PH can be further elevated in the coexistence of AMI and COPD. We hypothesized that PH may increase complications during hospitalization for reperfusion therapy for AMI and affect clinical outcomes in these patients. This study investigated the clinical impact of the presence of PH in AMI patients with or without COPD.

## 2 Methods

From November 2011 to December 2015, a total of 3009 patients with AMI from Chonnam National University Hospital (CNUH) were initially screened. Among them, we selected patients who underwent a pulmonary function test (PFT) and excluded the following cases:

1. patients who did not receive TTE and
2. patients whose RVSP value was not estimated on TTE.

**Figure 1:**
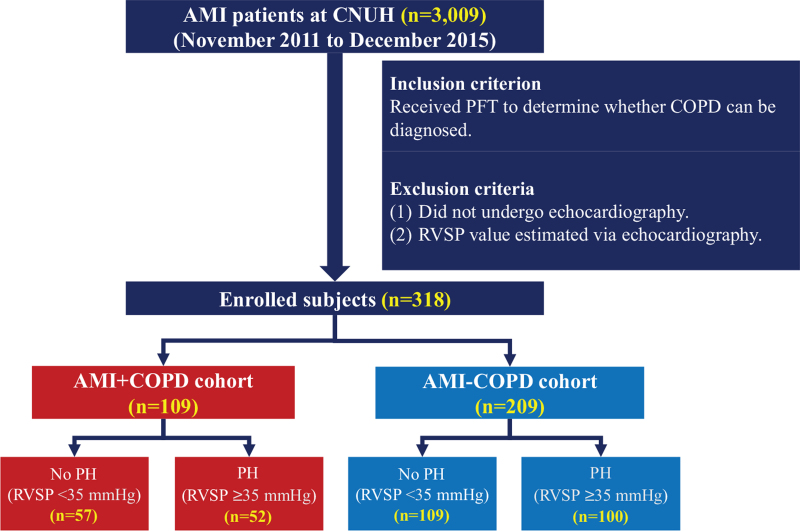
Study population flow chart. AMI = acute myocardial infarction; CNUH = Chonnam National University Hospital; COPD = chronic obstructive pulmonary disease; PFT = pulmonary function test; PH = pulmonary hypertension; RVSP = right ventricle systolic pressure.

After excluding these patients, a total of 318 AMI patients with COPD (AMI+COPD cohort) (n = 109) or without COPD (AMI-COPD cohort) (n = 209) were finally included in the study (Fig. 1). These patients were subdivided into 2 groups according to RVSP level (PH group [RVSP ≥35 mm Hg] and no PH group [RVSP <35 mm Hg]). The medical records of all patients were reviewed to clarify AMI and COPD during the study period. The data of baseline clinical characteristics, laboratory findings, prescribed medications, and results from TTE, coronary angiography, and PFT were reviewed by cardiologists and pulmonologists.

As emphasized by a number of guidelines,^[18,19]^ the term AMI means the necrosis of cardiomyocytes manifested by a rise and/or fall in cardiac biomarkers, with the clinical evidence of acute myocardial ischemia including at least one of the following:

1. clinical symptoms of myocardial ischemia;
2. new-onset ischemic change on an electrocardiogram;
3. development of pathologic Q-waves;
4. evidence from cardiovascular imaging modalities of new loss of viable myocardium or new regional wall motion abnormality in a pattern consistent with an ischemic etiology; and
5. identification of a coronary thrombus by angiography or autopsy.

All patients with AMI were categorized in accordance with the Killip classification at presentation (Killip class I: no HF; Killip class II: third heart sound and rales; Killip class III: overt pulmonary edema; and Killip class IV: cardiogenic shock).^[20]^ Among AMI, ST-segment elevation MI is defined as new-onset elevation of the ST segment in at least 2 continuous leads measuring >0.2 mV in precordial leads V1–3 or 0.1 mV in all other leads on a 12-lead surface electrocardiogram with a concomitant rise or fall in myocardial biomarkers.^[21]^ Both LV ejection fraction (LVEF) and RVSP were measured using two-dimensional TTE. RVSP was derived from the peak tricuspid regurgitant jet velocity (V) and right atrial pressure (RAP) with the modified Bernoulli equation (RVSP = 4 V^2^ + RAP).^[22]^ RAP was determined by the diameter of the inferior vena cava (IVC) and collapsibility of the IVC during inspiratory respiration.^[23]^ The Global Initiative for Chronic Obstructive Lung Disease guidelines were referenced in the process of defining COPD,^[24]^ and each measurement value of PFT was utilized to diagnose COPD.^[25]^

We investigated in-hospital clinical outcomes, which comprised in-hospital death, cardiogenic shock, new-onset HF, recurred non-fatal MI, stent thrombosis, cerebrovascular accidents, bleeding complications, cardiopulmonary resuscitation, and mechanical circulatory support such as intraaortic balloon pump and extracorporeal membrane oxygenation. Cardiogenic shock was defined as persistent hypotension (systolic blood pressure [SBP] <90 mm Hg for >30 minutes) and/or any clinical condition requiring vasopressors to achieve a SBP ≥90 mm Hg. New-onset HF was defined as the sudden appearance of clinical symptoms, such as dyspnea, fatigue, swelling in both lower extremities, rapid and/or irregular heartbeats, reduced exercise capacity, persistent cough and/or wheezes, and chest pain. We also investigated 1-year clinical outcomes of postdischarge survivors, including all-cause mortality, cardiac death, non-cardiac death, non-fatal MI, and rehospitalization. All-cause mortality was made up of both cardiac and non-cardiac deaths. Rehospitalization refers to any unplanned readmission due to clinical symptoms and/or signs of HF. We also defined a major adverse cardiac event (MACE) as the composite of all-cause mortality, non-fatal MI, and rehospitalization.

The study protocol was approved by the Institutional Review Board of CNUH (No. CNUH-2021-188), and the requirement for informed consent was waived because of the retrospective nature of this study. This study complied with the declaration of Helsinki.

The statistical analyses in this study were performed with SPSS version 25.0 (IBM Corp., Armonk, NY). We describe continuous variables as means ± standard deviations and discrete (categorical) variables as percentages (%) with numbers. Continuous variables were analyzed with Student *t* test and categorical variables with Pearson Chi-Squared test or Fisher exact test. All data results were considered statistically significant at a *P* value <.05. To explain the difference between the 2 groups, we developed a propensity score-weighted model using the inverse probability of treatment weighting (IPTW). This IPTW model included some clinical variables (male sex, age ≥65 years, SBP, diastolic blood pressure, pulse pressure ≥40 mm Hg, heart rate, Killip class III–IV, body mass index ≥25 kg/m^2^, prior medical history [hypertension, diabetes mellitus, dyslipidemia, prior MI, prior angina pectoris, prior heart failure, and prior cerebrovascular accident], smoking history, family history of coronary artery disease, final diagnosis, laboratory profiles [white blood cell count, neutrophil-to-lymphocyte ratio ≥2.5, hemoglobin {Hgb}, platelet count, creatinine, glucose, and troponin-I], procedural profiles [percutaneous coronary intervention, coronary artery bypass grafting, thrombolysis, glycoprotein IIb/IIIa inhibitors, and thrombus aspiration], echocardiographic profiles [LVEF <40%, regional wall motion index {RWMI}, left atrium {LA} diameter ≥40 mm, peak early transmitral inflow velocity {E}/mitral annulus velocity {E’} ratio >14, E’ <0.07 m/s, moderate or severe mitral regurgitation {MR}, and LV end-diastole diameter {LVEDD} ≥55 mm], PFT [forced expiratory volume in the first second {FEV1} and FEV1/forced vital capacity {FVC} >70%], and prescribed medications [aspirin, P2Y12 inhibitors, calcium channel blockers, beta-blockers, angiotensin-converting enzyme inhibitors or angiotensin receptor blockers, and statins]). Patients who had any missing data in these covariates or those with a follow-up interval after hospital discharge of zero days were excluded from the IPTW-adjusted analysis. In terms of 1-year clinical outcomes, the analysis of cumulative incidences was described using time-to-event data with the Kaplan–Meier method. Survival curves were compared using the log-rank test. Patient data was censored at the time of the event or at the final follow-up. Kaplan–Meier curves were drawn for the time of occurrence of clinical outcomes.

## 3 Results

The baseline characteristics are summarized in Tables 1 and 2. In the AMI-COPD cohort, the PH group was older, had a higher heart rate, and had higher proportions of female patients, Killip class III–IV, hypertension, diabetes mellitus, and non-smokers than the no PH group. The PH group was more anemic with a lower Hgb level and had higher troponin-I levels than the no PH group. In terms of echocardiographic profiles, the PH group had lower LVEF and E’ values but higher RWMI, LA diameter, E/E’ ratio, and LVEDD values. It also had a higher proportion of moderate or severe MR than the no PH group. In the AMI+COPD cohort, the PH group had higher proportions of Killip class III–IV, body mass index ≥25 kg/m^2^, hypertension, and ST-segment elevation MI as a final diagnosis. The PH group was more anemic with a lower Hgb level, and it had a higher LA diameter than the no PH group. Regarding prescribed medications, statins were more frequently prescribed in the no PH group than in the PH group. These between-group differences were well-balanced after IPTW adjustment (Table S1 and S2, Supplemental Digital Content).

**Table 1:** Demographic and general characteristics of patients.

	AMI without underlying COPD			AMI with underlying COPD		
Variables	RVSP <35 mmHg (n = 109)	RVSP ≥35 mmHg (n = 100)	*P* value	RVSP <35 mmHg (n = 57)	RVSP ≥35 mmHg (n = 52)	*P* value
Male sex	80 (73.4)	47 (47.0)	<.001	45 (78.9)	45 (86.5)	.297
Age, years	64.27 ± 9.85	70.49 ± 9.36	<.001	72.33 ± 8.69	72.73 ± 7.43	.799
Age ≥65 years	57 (52.3)	76 (76.0)	<.001	47 (82.5)	44 (84.6)	.762
Systolic blood pressure	124.68 ± 29.55	124.20 ± 25.75	.901	119.65 ± 22.68	124.42 ± 22.79	.276
Diastolic blood pressure	77.80 ± 17.92	77.60 ± 14.78	.931	75.09 ± 16.49	79.04 ± 14.45	.188
Pulse pressure ≥40 mmHg	101 (92.7)	91 (91.0)	.661	51 (89.5)	48 (92.3)	.745
Heart rate	78.47 ± 15.69	83.95 ± 19.13	.024	81.33 ± 16.06	87.12 ± 18.36	.082
Killip class III−IV	10 (9.2)	19 (19.0)	.040	6 (10.5)	15 (28.8)	.015
BMI, kg/m^2^						
BMI ≥25 kg/m^2^	34 (31.2)	27 (27.8)	.598	19 (33.9)	4 (8.0)	.002
Prior medical history						
Hypertension	58 (53.2)	72 (72.0)	.005	37 (64.9)	23 (44.2)	.030
Diabetes mellitus	35 (32.1)	46 (46.0)	.040	17 (29.8)	23 (44.2)	.119
Dyslipidemia	12 (11.0)	9 (9.0)	.629	2 (3.5)	0 (0.0)	.496
Prior MI	10 (9.2)	14 (14.0)	.274	9 (15.8)	10 (19.2)	.636
Prior angina	15 (13.8)	14 (14.0)	.960	6 (10.5)	9 (17.3)	.305
Prior HF	0 (0.0)	3 (3.0)	.108	4 (7.0)	1 (1.9)	.366
Prior CVA	14 (13.0)	9 (9.1)	.376	4 (7.0)	1 (1.9)	.366
Smoking history			.001			.081
Current smoker or ex-smoker	69 (63.3)	41 (41.0)		35 (61.4)	40 (76.9)	
Non-smoker	40 (36.7)	59 (59.0)		22 (38.6)	12 (23.1)	
Family history of CAD	8 (7.3)	2 (2.0)	.104	1 (1.8)	1 (1.9)	1.000
STEMI diagnosis	28 (25.7)	28 (28.0)	.706	12 (21.1)	20 (38.5)	.046

**Table 2:** Clinical characteristics of patients.

	AMI without underlying COPD			AMI with underlying COPD		
Variables	RVSP <35 mm Hg (n = 109)	RVSP ≥35 mm Hg (n = 100)	*P* value	RVSP <35 mm Hg (n = 57)	RVSP ≥35 mm Hg (n = 52)	*P* value
Laboratory profiles						
WBC, ×10^3^ /mm^3^	9.20 ± 3.45	12.29 ± 23.95	.184	10.30 ± 4.45	10.50 ± 3.02	.780
NLR ≥2.5	66 (60.6)	69 (69.0)	.202	40 (70.2)	42 (80.8)	.201
Hgb, g/dL	13.47 ± 2.15	12.19 ± 2.39	<.001	13.61 ± 1.88	12.24 ± 2.39	.001
Platelets, ×10^3^ /mm^3^	225.52 ± 62.08	218.00 ± 66.32	.398	216.26 ± 63.12	223.08 ± 79.59	.620
Glucose, mg/dL	157.22 ± 69.36	181.31 ± 106.65	.058	166.54 ± 80.68	177.71 ± 80.84	.475
Creatinine, mg/dL	0.98 ± 1.22	1.27 ± 1.13	.084	1.10 ± 1.48	1.21 ± 0.80	.649
Troponin-I, ng/mL	23.55 ± 43.96	40.34 ± 65.58	.034	29.82 ± 74.78	42.72 ± 62.31	.333
Procedural profiles						
PCI or CABG	91 (83.5)	79 (79.0)	.406	50 (87.7)	45 (86.5)	.854
PCI	81 (74.3)	76 (76.0)	.778	48 (84.2)	41 (78.8)	.470
CABG	10 (9.2)	3 (3.0)	.086	2 (3.5)	4 (7.7)	.422
Thrombolysis	0 (0.0)	0 (0.0)	-	1 (1.8)	0 (0.0)	1.000
GPIIb/IIIa inhibitors	11 (10.1)	11 (11.0)	.831	6 (10.5)	5 (9.6)	.875
Thrombus aspiration	5 (4.6)	4 (4.0)	1.000	1 (1.8)	0 (0.0)	1.000
Echocardiographic profiles						
LVEF <40%	10 (9.2)	23 (23.0)	.006	7 (12.3)	10 (19.2)	.318
RWMI	1.31 ± 0.34	1.53 ± 0.41	<.001	1.35 ± 0.34	1.57 ± 0.43	.004
LA diameter ≥40 mm	39 (36.1)	60 (60.0)	.001	17 (29.8)	30 (57.7)	.003
E/E’ ratio >14	25 (23.8)	57 (58.8)	<.001	16 (28.6)	24 (46.2)	.059
E’ <0.07 m/s	72 (68.6)	83 (84.7)	.007	43 (76.8)	42 (80.8)	.613
Moderate or severe MR	6 (5.5)	18 (18.0)	.005	6 (10.5)	5 (9.6)	.875
LVEDD ≥55 mm	14 (13.0)	25 (25.0)	.026	8 (14.0)	14 (26.9)	.094
RVSP	27.50 ± 4.89	44.22 ± 9.75	<.001	28.13 ± 4.52	42.99 ± 8.16	<.001
Pulmonary function test						
FEV1, L	2.45 ± 0.63	1.82 ± 0.70	<.001	1.64 ± 0.54	1.73 ± 0.53	.377
FEV1/FVC, %	80.86 ± 6.99	80.37 ± 7.11	.613	57.43 ± 10.91	58.42 ± 10.46	.631
Prescribed medications						
Aspirin	108 (99.1)	100 (100.0)	1.000	57 (100.0)	50 (96.2)	.225
P2Y12 inhibitors	107 (98.2)	99 (99.0)	1.000	57 (100.0)	52 (100.0)	-
CCB	16 (14.7)	11 (11.0)	.428	7 (12.3)	5 (9.6)	.764
BB	82 (75.2)	76 (76.0)	.897	36 (63.2)	35 (67.3)	.691
ACTi or ARB	82 (75.2)	82 (82.0)	.234	39 (68.4)	38 (73.1)	.594
Statins	101 (92.7)	86 (86.0)	.117	53 (93.0)	40 (76.9)	.018

The in-hospital clinical outcomes are summarized in Table 3. In the AMI-COPD cohort, the PH group had a higher proportion of new-onset HF. In the AMI+COPD cohort, the PH group had higher proportions of cardiogenic shock and new-onset HF. Based on the IPTW-adjusted data, cardiogenic shock was higher in the PH group in the AMI+COPD cohort (Table S3, Supplemental Digital Content).

**Table 3:** In-hospital clinical outcomes of the patients.

	AMI without underlying COPD			AMI with underlying COPD		
Variables	RVSP <35 mm Hg (n = 109)	RVSP ≥35 mm Hg (n = 100)	*P* value	RVSP <35 mm Hg (n = 57)	RVSP ≥35 mm Hg (n = 52)	*P* value
In-hospital death	1 (0.9)	3 (3.0)	.351	0 (0.0)	2 (3.8)	.225
Cardiogenic shock	12 (11.0)	11 (11.0)	.998	1 (1.8)	8 (15.4)	.013
New-onset HF	5 (4.6)	19 (19.0)	.001	6 (10.5)	13 (25.0)	.047
Recurred non-fatal MI	0 (0.0)	1 (1.0)	.478	0 (0.0)	1 (1.9)	.477
Stent thrombosis	0 (0.0)	1 (1.0)	.478	0 (0.0)	1 (1.9)	.477
CVA	6 (5.5)	3 (3.0)	.502	3 (5.3)	3 (5.8)	1.000
Bleeding complications						
Reduction in Hgb ≥5 g/dL	0 (0.0)	0 (0.0)	-	0 (0.0)	0 (0.0)	-
≥15% decrease in Hct	0 (0.0)	2 (2.0)	.228	0 (0.0)	0 (0.0)	-
Minor bleeding	3 (2.8)	2 (2.0)	1.000	1 (1.8)	5 (9.6)	.101
CPR	12 (11.0)	14 (14.0)	.513	3 (5.3)	8 (15.4)	.113
Mechanical circulatory support						
IABP	2 (1.8)	0 (0.0)	.499	0 (0.0)	2 (3.8)	.225
ECMO	1 (0.9)	1 (1.0)	1.000	0 (0.0)	1 (1.9)	.477

**Figure 2:**
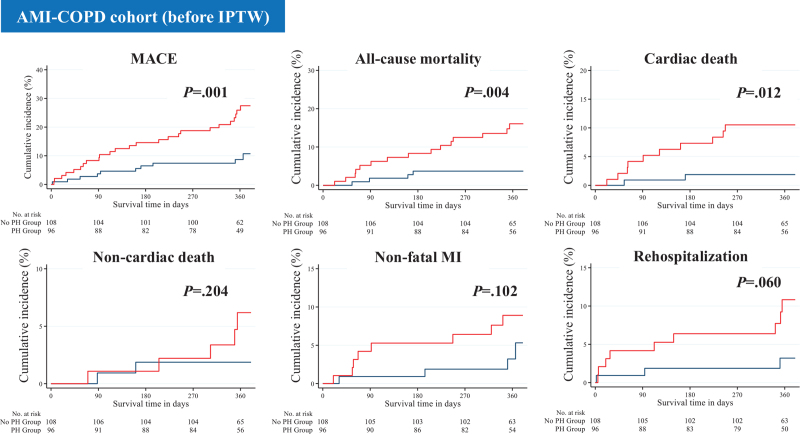
Event rates of long-term clinical outcomes for all the patients in the AMI-COPD cohort after 1-year follow-up (before IPTW). The figure shows the Kaplan–Meier curves for cumulative event rates according to the presence or absence of PH. Red curve indicates PH group, and blue curve indicates no PH group. AMI = acute myocardial infarction; COPD = chronic obstructive pulmonary disease; IPTW = inverse probability of treatment weighting; MI = myocardial infarction; PH = pulmonary hypertension.

**Figure 3:**
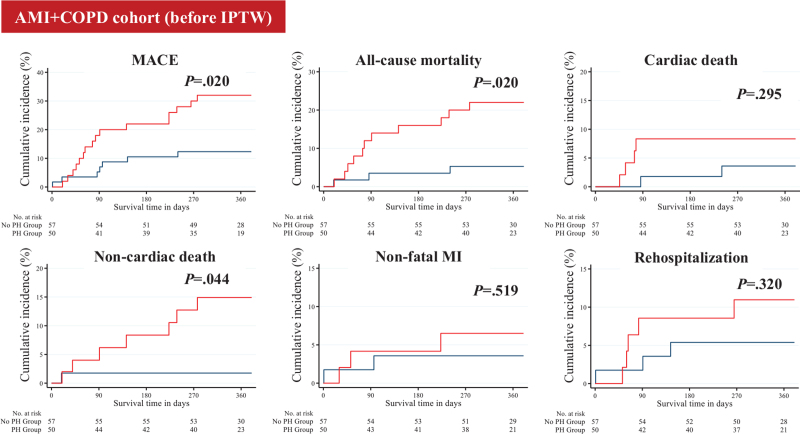
Event rates of long-term clinical outcomes for all the patients in the AMI+COPD cohort after 1-year follow-up (before IPTW). The figure shows the Kaplan–Meier curves for cumulative event rates according to the presence or absence of PH. Red curve indicates PH group, and blue curve indicates no PH group. AMI = acute myocardial infarction; COPD = chronic obstructive pulmonary disease; IPTW = inverse probability of treatment weighting; MI = myocardial infarction; PH = pulmonary hypertension.

The median follow-up period for the overall post-discharge survivors was 364 days. The 1-year clinical outcomes are summarized in Table 4 and Figures 2 and 3. In the AMI-COPD cohort, the PH group had higher proportions of all-cause mortality (17.71% [n = 17] vs 4.63% [n = 5], *P* = .004) and cardiac death than the no PH group (12.50% [n = 12] vs 2.78% [n = 3], *P* = .012). In the AMI+COPD cohort, the PH group had higher proportions of all-cause mortality (22.00% [n = 11] vs 5.26% [n = 3], *P* = .020) and non-cardiac death (14.00% [n = 7] vs 1.75% [n = 1], *P* = .044) than the no PH group. In both cohorts, the incidence of MACE was higher in the PH group than in the no PH group. After IPTW adjustment, these differences were statistically attenuated such that all variables were similar between both groups (Table S4 and S5, Supplemental Digital Content).

**Table 4:** One-year clinical outcomes of patients.

AMI without underlying COPD						
Outcomes	RVSP <35 mmHg (no PH group) (n = 108)	RVSP ≥35 mmHg (PH group) (n = 96)	Unadjusted analysis		IPTW-adjusted analysis	
			HR (95% CI)^∗^	*P* value	HR (95% CI)^∗∗^	*P* value
MACE	11/108	28/96	3.289 (1.596-6.778)	.001	2.011 (0.786-5.146)	.145
All-cause mortality	5/108	17/96	4.867 (1.637-14.469)	.004	2.762 (0.753-10.135)	.126
Cardiac death	3/108	12/96	6.832 (1.528-30.539)	.012	3.298 (0.636-17.098)	.155
Non-cardiac death	2/108	5/96	2.897 (0.562-14.934)	.204	1.911 (0.256-14.261)	.528
Non-fatal MI	4/108	10/96	2.676 (0.824-8.692)	.102	1.949 (0.511-7.429)	.328
Rehospitalization	3/108	9/96	3.502 (0.948-12.937)	.060	1.788 (0.406-7.876)	.442

AMI with underlying COPD						
Outcomes	RVSP <35 mmHg (no PH group) (n = 57)	RVSP ≥35 mmHg (PH group) (n = 50)	Unadjusted analysis		IPTW-adjusted analysis	
			HR (95% CI)^∗^	*P* value	HR (95% CI)^∗∗^	*P* value
MACE	7/57	16/50	2.883 (1.185-7.011)	.020	1.225 (0.437-3.435)	.700
All-cause mortality	3/57	11/50	4.528 (1.263-16.237)	.020	2.957 (0.766-11.411)	.116
Cardiac death	2/57	4/50	2.479 (0.454-13.540)	.295	1.954 (0.349-10.935)	.446
Non-cardiac death	1/57	7/50	8.623 (1.060-70.118)	.044	4.407 (0.506-38.410)	.179
Non-fatal MI	2/57	3/50	1.801 (0.301-10.788)	.519	0.498 (0.058-4.304)	.526
Rehospitalization	3/57	5/50	2.067 (0.494-8.655)	.320	0.754 (0.168-3.374)	.711

## 4 Discussion

We conducted a comparative analysis of the in-hospital and 1-year clinical outcomes among AMI patients with or without underlying COPD regarding the presence or absence of a PH diagnosis. We analyzed the data of 318 patients in CNUH, a single tertiary cardiovascular center. For in-hospital clinical outcomes, the PH group had high rates of new-onset HF regardless of the presence or absence of COPD, which was statistically attenuated post-IPTW. In the AMI+COPD cohort, however, cardiogenic shock was higher in the PH group than in the no PH group, which was also statistically maintained post-IPTW. In the 1-year clinical outcomes, some notable findings were brought to our attention. In both the AMI-COPD and AMI+COPD cohorts, the incidence rates of all-cause mortality were higher in the PH group than in the no PH group. Among the AMI-COPD cohort, the PH group had a higher incidence of cardiac death, whereas the PH group had a higher incidence of non-cardiac death in the AMI+COPD cohort. Although these 1-year clinical outcomes were merely the analytic results of unadjusted data and were statistically attenuated in IPTW-adjusted analysis, these trends were worthy of note.

Although statistically attenuated post-IPTW, the PH group had a higher proportion of new-onset HF regardless of the presence of a COPD diagnosis. In 1 clinical study conducted by Mutlak and colleagues, PH at index hospitalization is known to be a useful marker for the prediction of the development of HF.^[14]^ This correlation is somewhat consistent with our clinical findings. Meanwhile, the PH group showed higher rates of cardiogenic shock in the AMI+COPD cohort before and after IPTW. Interestingly, this trend was not observed in the AMI-COPD cohort. Given that not only AMI but also COPD can develop PH, it is plausible that mixed-etiology PH (i.e., groups 2 and 3 PH) may lead to and reinforce RV failure, which can reduce LV filling and ultimately result in the cardiogenic shock.^[26]^

In terms of 1-year clinical outcomes, both the AMI+COPD and AMI-COPD cohorts showed a higher incidence of all-cause mortality in the PH group than in the no PH group. One clinical study suggested that an increase in RVSP is associated with long-term mortality in patients with AMI.^[13]^ In the aforementioned study, some variables, including the grade of LV diastolic function, the severity of mitral regurgitation, age, and the wall motion score index, were different in accordance with the severity of RVSP. This finding is also consistent with our results, as our study showed that the PH group presented lower LVEF and E’ values but higher RWMI, LA diameter, E/E’ ratio, and LVEDD values, as well as higher prevalence of moderate or severe MR, than the no PH group. According to the American Society of Echocardiography guidelines, some of these echocardiographic variables, such as the E/E’ ratio, E’ value, and LA diameter, are known to be related to LV diastolic function.^[27]^ In addition, this study demonstrated LV diastolic function, which appeared to be associated with RVSP. Moreover, LV diastolic function may play an important role on all-cause mortality in patients with AMI. Some clinical studies have also showed that the assessment of diastolic function was a strong independent predictor of clinical outcomes of AMI.^[28–31]^ Recently, Bae and colleagues demonstrated that a higher degree of diastolic dysfunction in patients with AMI yields a higher the incidence of all-cause mortality, and the assessment of the degree of diastolic dysfunction may be a significant predictor of all-cause mortality in patients with AMI. Considering that the PH group tended to have a higher burden of abnormality in the LV structure and systolic and diastolic functions, our result is sufficiently predictable and clinically significant. Since this difference was attenuated in the IPTW-adjusted analysis, as expressed in Table 4 and Table S4 and S5, Supplemental Digital Content, the interpretation of these differences should be determined with caution.

In an unadjusted analysis, the PH group demonstrated a higher proportion of all-cause mortality than the no PH group. However, this difference was mainly driven by cardiac death in the AMI-COPD cohort, whereas it was mainly driven by non-cardiac death in the AMI+COPD cohort. This finding suggests that in the AMI+COPD cohort, PH was significantly associated with non-cardiac death rather than cardiac death. We should remember that patients with COPD can also develop PH, which is categorized as group 3 PH according to the World Health Organization classification.^[32]^ Moreover, PH can also affect the clinical outcomes of patients with COPD. In patients with COPD, PH acts as a strong predictor of mortality,^[33]^ as COPD patients with PH have increased morbidity and adverse events.^[34]^ In advanced COPD, PH is known as one of the most frequent clinical manifestations, and COPD has a worse outcome if accompanied by PH.^[35]^ Although RV failure is one of the well-known pathophysiological explanations for the mortality, there is a paucity of information about the real nature of all-cause mortality among these patients. Because the high proportion of non-cardiac mortality found in the PH group is a notable part of this study, we additionally examined a total of 8 patients in the AMI+COPD cohort (7 in the PH group and 1 in the no PH group) who were deceased from non-cardiac etiologies (Table S6, Supplemental Digital Content). Among them, 5 patients were deceased due to pulmonary diseases (1 by asphyxia due to hemoptysis, 2 by primary lung malignancy, 1 by pneumosepsis, and 1 by respiratory arrest), which indicates that pulmonary diseases may account for a considerable proportion of non-cardiac mortality in the AMI+COPD cohort. Although it is well-known that secondary PH is associated with mortality in the AMI population, it can be inferred that a considerable number of non-cardiac deaths due to any pulmonary etiology also exists in the AMI+COPD cohort.

Through literature review, we identified distinct studies about the clinical impact of PH on AMI or COPD.^[13,14,34,36,37]^ However, limited information exists regarding the effect of PH on clinical outcomes when AMI and COPD co-exist. Taking into consideration that the elevation of PAP in each of the 2 diseases is related to the occurrence of adverse events, it is plausible that the presence of PH may be associated with complications in index hospitalization patients with the coexistence of these 2 diseases. To the best of our knowledge, this study is the first investigation to elucidate the association of PH (RVSP) with in-hospital and one-year clinical outcomes in patients with both AMI and COPD. We expect that this study will provide new insights into the clinical impact of PH on outcomes in patients with co-existing AMI and COPD.

However, our study has some key limitations.

First, this was a small-sized, single-center observational study. Due to small sample size, this study is likely to have low statistical power. Moreover, all differences in 1-year clinical outcomes were not maintained after IPTW adjustment. Although this study can be regarded as a hypothesis-generating study, our results are difficult to generalize. Second, all patients underwent Doppler TTE for the assessment of RVSP and evaluation of PH. Although TTE is known to be suitable with adequate reliability to establish a non-invasive diagnosis of PH,^[38]^ the accuracy of this modality in identifying PH has been challenged,^[39]^ and RHC is still the accepted gold standard for its diagnosis. Unfortunately, the patients in this study were not tested using RHC. Third, although this study included a history of prior HF in baseline characteristics, our study did not present any available data on pre-existing diastolic dysfunction or valvular heart disease. There was also no measurement of baseline PAP before hospitalization. Hence, it is impossible to know if PH observed in patients after AMI was due to chronic pulmonary hypertension (group 3 PH) or an acute elevation of pulmonary pressure due to dysfunction of the left heart (group 2 PH). Moreover, our study did not mention the baseline functional status of patients, such as exercise tolerance and the 6-minute walk test.

Fourth, since this was a non-randomized study, statistical problems arising from selection bias are inevitable. Moreover, since a large proportion of patients with AMI did not undergo routine PFT during hospitalization, only 318 of 3009 patients with AMI were finally enrolled into the analysis of this study, which also may have caused selection bias. Although propensity score weighting was conducted to minimize the selection bias, a multicenter randomized controlled trial is needed in the future. In addition, because factors which confound the relationship between PH and clinical outcomes may theoretically exist, careful consideration of potential confounders between is necessary in interpreting the results of the present study.

Despite these limitations, our results highlight that in-hospital and 1-year clinical outcomes are somewhat different in both the PH and no PH groups among AMI patients with or without COPD. Although statistically insignificant in IPTW-adjusted analysis, PH may be associated with new-onset HF in these patients. PH is also associated with cardiogenic shock in patients with AMI and COPD, which is consistent with the result of the post-IPTW analysis. In addition, although it was statistically attenuated in the IPTW-adjusted analysis, PH may be associated with 1-year all-cause mortality in both the AMI-COPD and AMI+COPD cohorts, perhaps by a different etiology for each. We have concluded that clinical research is required to elucidate these findings in a larger population in the future.

## Acknowledgments

We sincerely thank Cho-Hee Hwang, a biostatistician, for participating in the statistical analysis of this study.

## Author contributions

**Conceptualization:** Seok Oh.

**Data curation:** Seok Oh, Kyung Hoon Cho, Min Chul Kim, Doo Sun Sim, Young Joon Hong.

**Formal analysis:** Seok Oh.

**Investigation:** Seok Oh, Ju Han Kim.

**Software:** Seok Oh.

**Writing – original draft:** Seok Oh.

**Writing – review & editing:** Ju Han Kim, Kyung Hoon Cho, Min Chul Kim, Doo Sun Sim, Young Joon Hong, Youngkeun Ahn, Myung Ho Jeong.

## Supplementary Material

Supplemental Digital Content

## Supplementary Material

Supplemental Digital Content

## Supplementary Material

Supplemental Digital Content

## Supplementary Material

Supplemental Digital Content

## Supplementary Material

Supplemental Digital Content

## Supplementary Material

Supplemental Digital Content
