# Supplementary material for: Clinical impact of pulmonary hypertension on the outcomes of acute myocardial infarction patients with or without chronic obstructive pulmonary disease
Source: Medicine (Baltimore). 2022 Jan 21;101(3):e28627. doi: 10.1097/MD.0000000000028627 (PMC8772642; doi:10.1097/MD.0000000000028627)
Supplement: Supplemental Digital Content [file medi-101-e28627-s001.doc]

**Supplemental Digital Content 1**. Demographic and general characteristics of patients after IPTW

| **Variables** | **AMI without underlying COPD** | | | **AMI with underlying COPD** | | |
| --- | --- | --- | --- | --- | --- | --- |
|  | RVSP <35 mmHg (n=179) | RVSP ≥35 mmHg (n=217) | *P* value | RVSP <35 mmHg (n=96) | RVSP ≥35 mmHg (n=84) | *P* value |
| **Male sex** | 124 (69.0) | 148 (68.0) | .924 | 81 (84.4) | 76 (90.2) | .366 |
| **Age, years** | 66.58±9.48 | 63.62±11.83 | .454 | 71.89±8.15 | 71.40±7.50 | .794 |
| **Age ≥65 years** | 111 (61.7) | 104 (47.9) | .303 | 84 (87.2) | 67 (80.3) | .426 |
| **Systolic blood pressure** | 123.93±29.25 | 130.73±23.78 | .268 | 115.85±22.40 | 122.30±22.04 | .181 |
| **Diastolic blood pressure** | 76.66±17.52 | 80.77±13.41 | .251 | 72.33±15.82 | 76.73±14.50 | .182 |
| **Pulse pressure ≥40 mmHg** | 169 (94.2) | 204 (94.1) | .971 | 87 (91.0) | 78 (93.7) | .580 |
| **Heart rate** | 74.58±20.05 | 78.93±15.31 | .431 | 81.47±15.93 | 85.11±17.39 | .293 |
| **Killip class III-IV** | 18 (10.3) | 23 (10.8) | .925 | 13 (14.0) | 20 (23.3) | .328 |
| **BMI, kg/m^2^** |  |  |  |  |  |  |
| **BMI ≥25 kg/m^2^** | 52 (29.3) | 58 (26.6) | .803 | 22 (22.7) | 12 (13.7) | .403 |
| **Prior medical history** |  |  |  |  |  |  |
| **Hypertension** | 118 (65.9) | 114 (52.6) | .331 | 51 (53.2) | 40 (47.1) | .638 |
| **Diabetes mellitus** | 60 (33.5) | 97 (44.8) | .384 | 25 (26.5) | 38 (45.6) | .097 |
| **Dyslipidemia** | 16 (9.0) | 16 (7.3) | .698 | 2 (2.1) | 0 (0.0) | .214 |
| **Prior MI** | 13 (7.0) | 19 (8.7) | .689 | 13 (14.0) | 16 (18.5) | .567 |
| **Prior angina** | 23 (13.0) | 18 (8.5) | .385 | 8 (8.7) | 16 (18.5) | .169 |
| **Prior HF** | 0 (0.0) | 4 (1.7) | .188 | 7 (7.1) | 2 (2.1) | .248 |
| **Prior CVA** | 19 (10.6) | 29 (13.5) | .704 | 6 (6.6) | 2 (2.1) | .278 |
| **Smoking history** |  |  | .620 |  |  | .295 |
| **Current smoker or ex-smoker** | 113 (63.1) | 123 (56.6) |  | 70 (72.8) | 69 (81.9) |  |
| **Non-smoker** | 66 (36.9) | 94 (43.4) |  | 26 (27.2) | 15 (18.1) |  |
| **Family history of CAD** | 6 (3.4) | 1 (0.5) | .050 | 1 (1.2) | 3 (3.2) | .487 |
| **STEMI diagnosis** | 58 (32.6) | 42 (19.4) | .227 | 30 (31.1) | 29 (34.6) | .790 |

Values are presented as a number (percentage) for categorical values and means±standard deviations for continuous variables.

AMI = acute myocardial infarction; BMI = body mass index; CAD = coronary artery disease; COPD = chronic obstructive pulmonary disease; CVA = cerebrovascular accident; HF = heart failure; IPTW = inverse probability of treatment weighting; MI = myocardial infarction; RVSP = right ventricle systolic pressure; STEMI = ST-segment elevation myocardial infarction.
