# Supplementary material for: Clinical impact of pulmonary hypertension on the outcomes of acute myocardial infarction patients with or without chronic obstructive pulmonary disease
Source: Medicine (Baltimore). 2022 Jan 21;101(3):e28627. doi: 10.1097/MD.0000000000028627 (PMC8772642; doi:10.1097/MD.0000000000028627)
Supplement: Supplemental Digital Content [file medi-101-e28627-s002.doc]

**Supplemental Digital Content 2**. Clinical characteristics of patients after IPTW

|  |  |  |  |  |  |  |
| --- | --- | --- | --- | --- | --- | --- |
| **Variables** | **AMI without underlying COPD** | | | **AMI with underlying COPD** | | |
|  | RVSP <35 mmHg (n=179) | RVSP ≥35 mmHg (n=217) | *P* value | RVSP <35 mmHg (n=96) | RVSP ≥35 mmHg (n=84) | *P* value |
| **Laboratory profiles** |  |  |  |  |  |  |
| **WBC, ×10^3^ /mm^3^** | 9.05±2.87 | 9.76±16.52 | .607 | 10.76±4.91 | 10.66±3.03 | .917 |
| **NLR ≥2.5** | 114 (63.9) | 110 (51.0) | .345 | 75 (78.0) | 66 (78.6) | .951 |
| **Hgb, g/dL** | 13.16±2.05 | 13.03±2.27 | .813 | 13.56±1.78 | 12.91±2.61 | .243 |
| **Platelets, ×10^3^ /mm^3^** | 221.44±64.79 | 222.19±62.57 | .953 | 220.56±63.15 | 221.69±75.49 | .944 |
| **Glucose, mg/dL** | 162.54±66.54 | 171.98±88.05 | .661 | 165.39±77.60 | 172.31±74.81 | .643 |
| **Creatinine, mg/dL** | 1.10±1.37 | 1.11±0.91 | .985 | 1.56±2.58 | 1.17±0.73 | .531 |
| **Troponin-I, ng/mL** | 28.62±45.76 | 25.00±51.06 | .700 | 37.88±70.84 | 42.90±59.44 | .746 |
| **Procedural profiles** |  |  |  |  |  |  |
| **PCI or CABG** | 153 (85.5) | 192 (88.5) | .374 | 89 (92.7) | 77 (91.7) | .795 |
| **PCI** | 140 (78.3) | 167 (76.8) | .883 | 86 (89.5) | 72 (85.7) | .545 |
| **CABG** | 13 (7.5) | 25 (11.4) | .606 | 3 (3.5) | 5 (6.3) | .521 |
| **Thrombolysis** | 0 (0.0) | 0 (0.0) | - | 1 (1.1) | 0 (0.0) | .368 |
| **GPIIb/IIIa inhibitors** | 34 (19.1) | 16 (7.4) | .177 | 12 (12.4) | 12 (14.1) | .856 |
| **Thrombus aspiration** | 6 (3.3) | 5 (2.5) | .709 | 2 (2.1) | 0 (0.0) | .359 |
| **Echocardiographic profiles** |  |  |  |  |  |  |
| **LVEF <40%** | 16 (9.0) | 25 (11.7) | .592 | 14 (14.2) | 11 (12.9) | .855 |
| **RWMI** | 1.38±0.34 | 1.46±0.35 | .224 | 1.40±0.35 | 1.54±0.41 | .109 |
| **LA diameter ≥40 mm** | 83 (46.3) | 77 (35.3) | .382 | 31 (32.4) | 41 (48.9) | .169 |
| **E/E’ ratio >14** | 66 (36.9) | 66 (30.5) | .601 | 26 (26.9) | 32 (38.1) | .298 |
| **E’ <0.07 m/s** | 137 (76.4) | 184 (84.7) | .287 | 66 (68.6) | 59 (70.3) | .899 |
| **Moderate or severe MR** | 10 (5.8) | 19 (8.6) | .469 | 13 (13.6) | 6 (7.4) | .339 |
| **RVSP** | 27.77±4.66 | 40.49±7.40 | **<.001** | 27.76±4.36 | 41.88±8.01 | **<.001** |
| **LVEDD ≥55 mm** | 23 (12.9) | 47 (21.5) | .313 | 15 (15.5) | 20 (23.5) | .387 |
| **Pulmonary function test** |  |  |  |  |  |  |
| **FEV1, L** | 2.17±0.69 | 2.46±1.07 | .448 | 1.85±0.68 | 1.76±0.50 | .658 |
| **FEV1/FVC, %** | 79.63±6.85 | 79.97±6.01 | .811 | 59.02±9.12 | 57.59±9.80 | .503 |
| **Prescribed medications** |  |  |  |  |  |  |
| **Aspirin** | 177 (98.9) | 217 (100.0) | .282 | 96 (100.0) | 80 (95.6) | .164 |
| **P2Y12 inhibitors** | 176 (98.0) | 215 (99.1) | .544 | 96 (100.0) | 84 (100.0) | 1.000 |
| **CCB** | 22 (12.3) | 55 (25.6) | .280 | 9 (9.9) | 11 (12.5) | .703 |
| **BB** | 138 (76.9) | 182 (84.1) | .331 | 66 (68.2) | 59 (70.1) | .862 |
| **ACEi or ARB** | 141 (78.7) | 170 (78.3) | .966 | 68 (70.4) | 65 (77.7) | .468 |
| **Statins** | 164 (91.7) | 157 (72.4) | .073 | 86 (89.4) | 70 (84.0) | .537 |

Values are presented as a number (percentage) for categorical values and means±standard deviations for continuous variables.

ACEi = angiotensin-converting enzyme inhibitor; AMI = acute myocardial infarction; ARB = angiotensin receptor blocker; BB = beta-blocker; CABG = coronary artery bypass grafting; CCB = calcium channel blocker; COPD = chronic obstructive pulmonary disease; E = peak early transmitral inflow velocity; E’ = mitral annulus velocity; FEV1 = forced expiratory volume in the first second; FVC = forced vital capacity; GPIIb/IIIa = glycoprotein IIb/IIIa; Hgb = hemoglobin; IPTW = inverse probability of treatment weighting; LA = left atrium; LVEDD = left ventricular end-diastole dimension; LVEF = left ventricular ejection fraction; MR = mitral regurgitation; NLR = neutrophil-to-lymphocyte ratio; PCI = percutaneous coronary intervention; RVSP = right ventricle systolic pressure; WBC = white blood cell.
