# Supplementary material for: Clinical impact of pulmonary hypertension on the outcomes of acute myocardial infarction patients with or without chronic obstructive pulmonary disease
Source: Medicine (Baltimore). 2022 Jan 21;101(3):e28627. doi: 10.1097/MD.0000000000028627 (PMC8772642; doi:10.1097/MD.0000000000028627)
Supplement: Supplemental Digital Content [file medi-101-e28627-s003.doc]

**Supplemental Digital Content 3**. In-hospital clinical outcomes of patients after IPTW adjustment

| **Variables** | **AMI without underlying COPD** | | | **AMI with underlying COPD** | | |
| --- | --- | --- | --- | --- | --- | --- |
|  | RVSP <35 mmHg (n=179) | RVSP ≥35 mmHg (n=217) | *P* value | RVSP <35 mmHg (n=96) | RVSP ≥35 mmHg (n=84) | *P* value |
| **In-hospital death** | 1 (0.6) | 4 (1.9) | .323 | 0 (0.0) | 3 (2.8) | .145 |
| **Cardiogenic shock** | 38 (21.4) | 18 (8.3) | .155 | 1 (1.1) | 9 (11.2) | **<.001** |
| **New-onset HF** | 7 (4.1) | 22 (10.3) | .111 | 8 (8.7) | 15 (17.4) | .161 |
| **Recurred non-fatal MI** | 0 (0.0) | 1 (0.5) | .393 | 0 (0.0) | 1 (1.2) | .295 |
| **Stent thrombosis** | 0 (0.0) | 1 (0.5) | .393 | 0 (0.0) | 1 (1.2) | .295 |
| **CVA** | 10 (5.5) | 5 (2.1) | .224 | 6 (6.3) | 5 (5.8) | .915 |
| **Bleeding complications** |  |  |  |  |  |  |
| **Reduction in Hgb ≥5 g/dL** | 0 (0.0) | 0 (0.0) | 1.000 | 0 (0.0) | 0 (0.0) | 1.000 |
| **≥15% decrease in Hct** | 0 (0.0) | 4 (1.6) | .261 | 0 (0.0) | 0 (0.0) | 1.000 |
| **Minor bleeding** | 5 (2.9) | 17 (7.7) | .374 | 2 (2.4) | 7 (7.7) | .269 |
| **CPR** | 14 (7.6) | 36 (16.8) | .192 | 4 (4.2) | 9 (10.8) | .153 |
| **Mechanical circulatory support** |  |  |  |  |  |  |
| **IABP** | 2 (1.1) | 0 (0.0) | .282 | 0 (0.0) | 4 (4.4) | .135 |
| **ECMO** | 0 (0.0) | 2 (0.7) | .391 | 0 (0.0) | 1 (1.3) | .294 |
| **Total length of hospital stay** | 11.50±10.73 | 12.26±8.72 | .815 | 8.94±8.27 | 11.83±16.43 | .257 |

Values are presented as a number (percentage) for categorical values and means±standard deviations for continuous variables.

AMI = acute myocardial infarction; COPD = chronic obstructive pulmonary disease; CPR = cardiopulmonary resuscitation; CVA = cerebrovascular accident; ECMO = extracorporeal mechanical oxygenation; Hct = hematocrit; HF = heart failure; Hgb = hemoglobin; IABP = intra-aortic balloon pump; ICD = implantable cardioverter defibrillator; IPTW = inverse probability of treatment weighting; MI = myocardial infarction; RVSP = right ventricle systolic pressure
