# Supplementary material for: Clinical impact of pulmonary hypertension on the outcomes of acute myocardial infarction patients with or without chronic obstructive pulmonary disease
Source: Medicine (Baltimore). 2022 Jan 21;101(3):e28627. doi: 10.1097/MD.0000000000028627 (PMC8772642; doi:10.1097/MD.0000000000028627)
Supplement: Supplemental Digital Content [file medi-101-e28627-s004.doc]

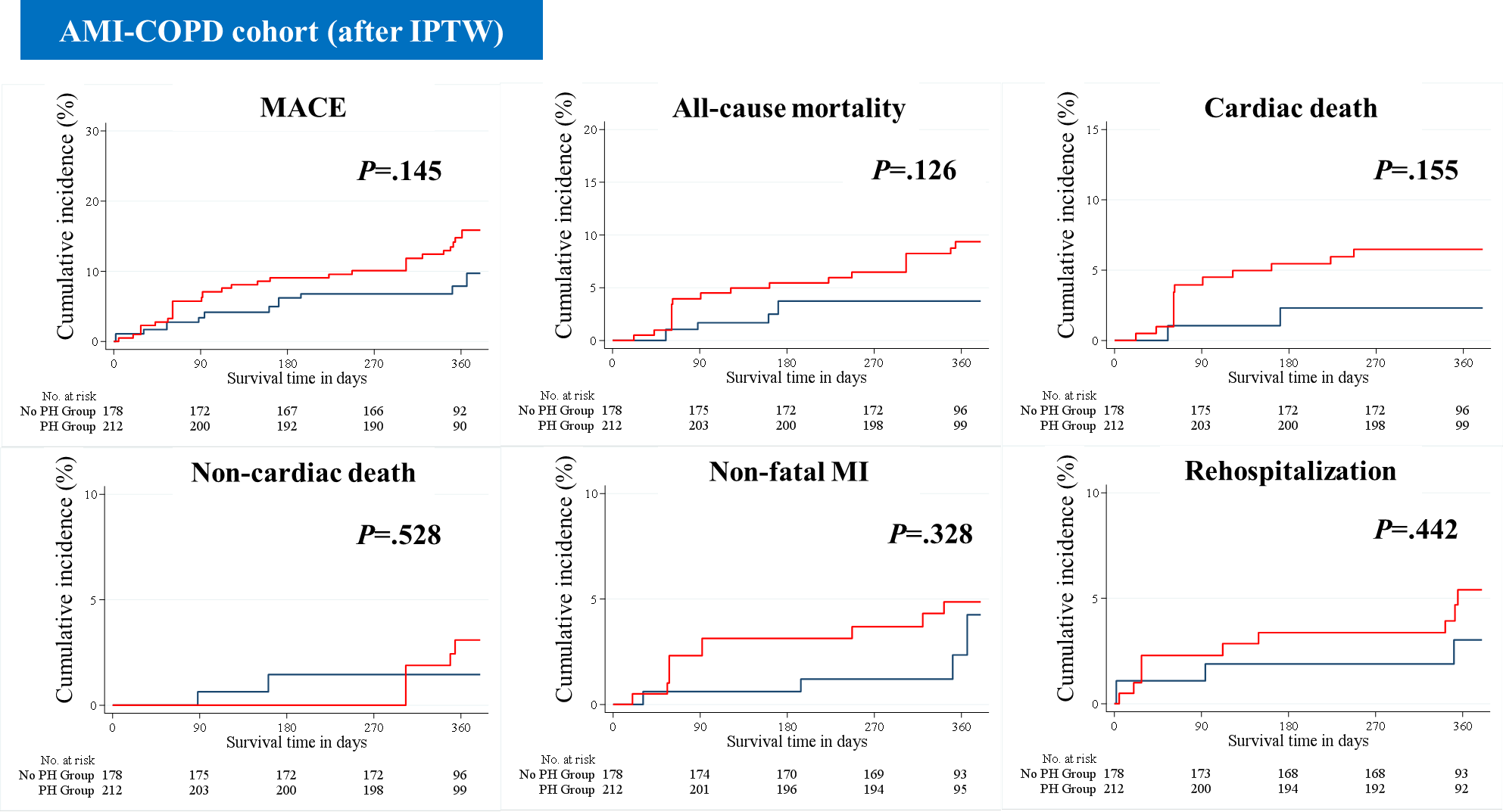
**Supplemental Digital Content 4.** Figure that illustrates event rates of long-term clinical outcomes for all the patients in the AMI-COPD cohort after one-year follow-up (after IPTW). The figure shows the Kaplan–Meier curves for cumulative event rates according to the presence or absence of PH. Red curve indicates PH group, and blue curve indicates no PH group. AMI = acute myocardial infarction; COPD = chronic obstructive pulmonary disease; IPTW = inverse probability of treatment weighting; MI = myocardial infarction; PH = pulmonary hypertension.
