# Supplementary material for: Clinical impact of pulmonary hypertension on the outcomes of acute myocardial infarction patients with or without chronic obstructive pulmonary disease
Source: Medicine (Baltimore). 2022 Jan 21;101(3):e28627. doi: 10.1097/MD.0000000000028627 (PMC8772642; doi:10.1097/MD.0000000000028627)
Supplement: Supplemental Digital Content [file medi-101-e28627-s006.doc]

**Supplemental** **Digital Content 6.** Detailed information about patients who died from non-cardiac etiologies in the AMI+COPD cohort

|  | **Group** | **Date of death** | **Cause of death** |
| --- | --- | --- | --- |
| **Case 1** | PH group | October 2, 2014 | Sepsis, acute kidney injury |
| **Case 2** | PH group | May 24, 2014 | Asphyxia due to hemoptysis (respiratory arrest) |
| **Case 3** | PH group | September 10, 2012 | Gastrointestinal bleeding |
| **Case 4** | PH group | April 11, 2013 | Pancreatic cancer |
| **Case 5** | PH group | April 2, 2012 | Primary lung malignancy |
| **Case 6** | PH group | July 17, 2012 | Respiratory arrest. |
| **Case 7** | PH group | February 10, 2013 | Primary lung malignancy |
| **Case 8** | No PH group | September 14, 2015 | Pneumosepsis |

AMI = acute myocardial infarction; COPD = chronic obstructive pulmonary disease
